# Electrospun polycaprolactone (PCL)-amnion nanofibrous membrane prevents adhesions and promotes nerve repair in a rat model of sciatic nerve compression

**DOI:** 10.1371/journal.pone.0244301

**Published:** 2020-12-18

**Authors:** Ruiyi Dong, Chunjie Liu, Siyu Tian, Jiangbo Bai, Kunlun Yu, Lei Liu, Dehu Tian

**Affiliations:** 1 Department of Orthopedics, Cangzhou Integrated Traditional Chinese and Western Medicine Hospital, Cangzhou, Hebei, China; 2 Department of Orthopedics, Tangshan Workers Hospital, Tangshan, Hebei, China; 3 Department of Hand Surgery, The Third Affiliated Hospital of Hebei Medical University, Shijiazhuang, Hebei, China; 4 Department of Orthopedics, Changping District Hospital, Beijing, China; Università degli Studi della Campania, ITALY

## Abstract

Adhesion and scarring after neural surgery are detrimental to nerve regeneration and functional recovery. Amniotic membranes have been used in tissue repair due to their immunogenicity and richness in cytokines. In this study, an electrospun polycaprolactone (PCL)-amnion nanofibrous membrane was prepared for the treatment of sciatic nerve compression in a rat model. The effects of the PCL-amnion nanofibrous membrane on the prevention of adhesion formation and nerve regeneration were evaluated using electrophysiology and histological analyses. Compared with the medical chitosan hydrogel dressing, the PCL-amnion nanofibrous membrane significantly reduced peripheral nerve adhesion and promoted the rapid recovery of nerve conduction. Moreover, the immunohistochemical analysis identified more Schwann cells and less pro-inflammatory M1 macrophages in the PCL-amnion group. Western blot and RT-PCR results showed that the expression levels of type-Ⅰ and Ⅲ collagen in the PCL-treated rats were half of those in the control group after 12 weeks, while the expression level of nerve growth factor was approximately 3.5 times that found in the rats treated with medical chitosan hydrogel. In summary, electrospun PCL-amnion nanofibrous membranes can effectively reduce adhesion after neural surgery and promote nerve repair and regeneration. The long-term retention *in vivo* and sustained release of cytokines make PCL-amnion a promising biomaterial for clinical application.

## 1. Introduction

Chronic nerve compression (CNC) refers to the nerve dysfunction caused by chronic compression of a specific area of the peripheral nerves, resulting in muscle atrophy and motor dysfunction [1]. In the early stage, the nerves become slightly ischemic and the blood-nerve barrier breaks down. With the increase of endoneurial fluid pressure, edema appears in the thickened endoneurium and perineurium, leading to segmental demyelination of the nerve fibers [2, 3]. Increasing pressure leads to aggravated and extensive ischemia, and the nerves adhere closely to the surrounding tissues, resulting in new compression and Wallerian degeneration [4].

Neurolysis relieves nerve compression and restores limb function [5]. However, the main causes of the failure of peripheral nerve surgery are adhesion, scarring, hemorrhage, and damage to the nerve as well as the surrounding tissues [6]. To prevent adhesion and scar formation, a variety of surgical methods have been developed, such as nerve transfer [7], fascial fat transplantation [8], venous encapsulation [9], and muscle flap transplantation [10]. However, even fine surgical techniques cannot completely rule out postoperative adhesions, and the tissues may still need to be obtained from different body parts [11]. Many natural and synthetic polymers are used to prevent adhesion, such as polyethylene films, silica gel patches [12], hyaluronic acid [13], and collagen [14]. Among these, non-biological materials are easy to obtain or prepare and have good mechanical properties, but they are usually not fully absorbed, resulting in residues of foreign substances [15]. Their lack of permeability also hinders the nutrient exchange of nerve cells and delays the recovery of nerve function [16].

Amniotic membrane has been widely used in the treatment of abdominal and pelvic adhesions, ophthalmic surgery, and chronic skin ulcers, due to their low immunogenicity [17, 18]. The amnion has no membranous structure of blood vessels, lymph, or nerves [19], and the amniotic epithelial cells can secrete glial cell-derived neurotrophic factor, epidermal growth factor, and transforming growth factor [20]. The amnion has been found to significantly alleviate inflammatory reactions and promote the recovery of nerve function; however, the amniotic membrane begins to be absorbed after 4 weeks and is completely absorbed after 12 weeks [21]. Moreover, the thickness of the amniotic membrane is only 0.02–0.5 mm, and its low mechanical strength and poor anti-extrusion capacity do not provide space for nerve regeneration [22, 23].

Polycaprolactone (PCL) is a polymer synthesized by the polymerization of ε-caprolactone monomers, catalyzed by a metal anion complex. It has good biocompatibility and biodegradability and has been applied in artificial skin materials, implanted bone-screws, and intravascular stents [24]. In this study, a rat model of sciatic nerve compression was established. An electrospun polycaprolactone (PCL)-amnion nanofibrous membrane was prepared, to improve the mechanical strength of the amniotic membrane, and applied to the compressed nerves. Through electrophysiology and histological studies, it was confirmed that PCL-amnion nanofibrous membranes can prevent adhesion formation and enhance nerve regeneration.

## 2. Materials and methods

### 2.1 Preparation of electron PCL-amnion nanofibrous membranes

The electron PCL-amnion nanofibrous membranes were designed and prepared following our previous reports. Briefly, 1 g PCL (average Mn-80,000, Sigma-Aldrich, US) and 0.5g gelatin from porcine skin (Vetec reagent grade, Type A, Sigma-Aldrich, US) were completely dissolved in 10 mL of hexafluoroisopropanol (>98%, Shanghai Nortel Co., Ltd., China) at room temperature. The mixture was stirred and defoamed by a magnetic rod overnight. When the room temperature was 25°C and humidity was 60%, the electrospinning device was connected. The electrospinning solution was placed into a 2 mL syringe with a needle diameter of 0.7 mm. The flow rate of the solution was adjusted to 1.0 mL/h, the voltage was set at 13 kV and the receiving distance was set at 15 cm. The PCL nanofibers were received on the two surfaces of freeze-dried amnions. The nanofiber membrane was dried overnight in a vacuum container. The freeze-dried amniotic epithelial cells were able to be closely arranged with an intact structure, flat surface, slightly dry shrinkage and clear intercellular junction structure, while the nanofibers were cylindrical, continuous, smooth and beadles. The average diameter of the nanofibers was 475.4 ± 147.5 nm, and the porosity was 77.0% ± 10.4%. The hydrophilic angles of freeze-dried amnion and nanofiber membrane were 51.18° ± 2.72° and 59.44° ± 4.15°, respectively. The maximum modulus of elasticity of the nanofiber membrane was higher than that of the freeze-dried amnion, and the tensile strength and toughness of the nanofiber membrane were higher than those of the freeze-dried amnion [25]. The nanofibrous membranes were sterilized with UV radiation before use [26].

### 2.2 Construction of the rat model of sciatic nerve compression

All animal experiments were in accordance with the guidance for the care and use of laboratory animals in China and approved by the Ethical Committee of the Third Hospital of Hebei Medical University. Adult male SD rats with body weights of 200–250 grams were provided by the Animal Experimental Center of Hebei Medical University.

**Fig 1:**
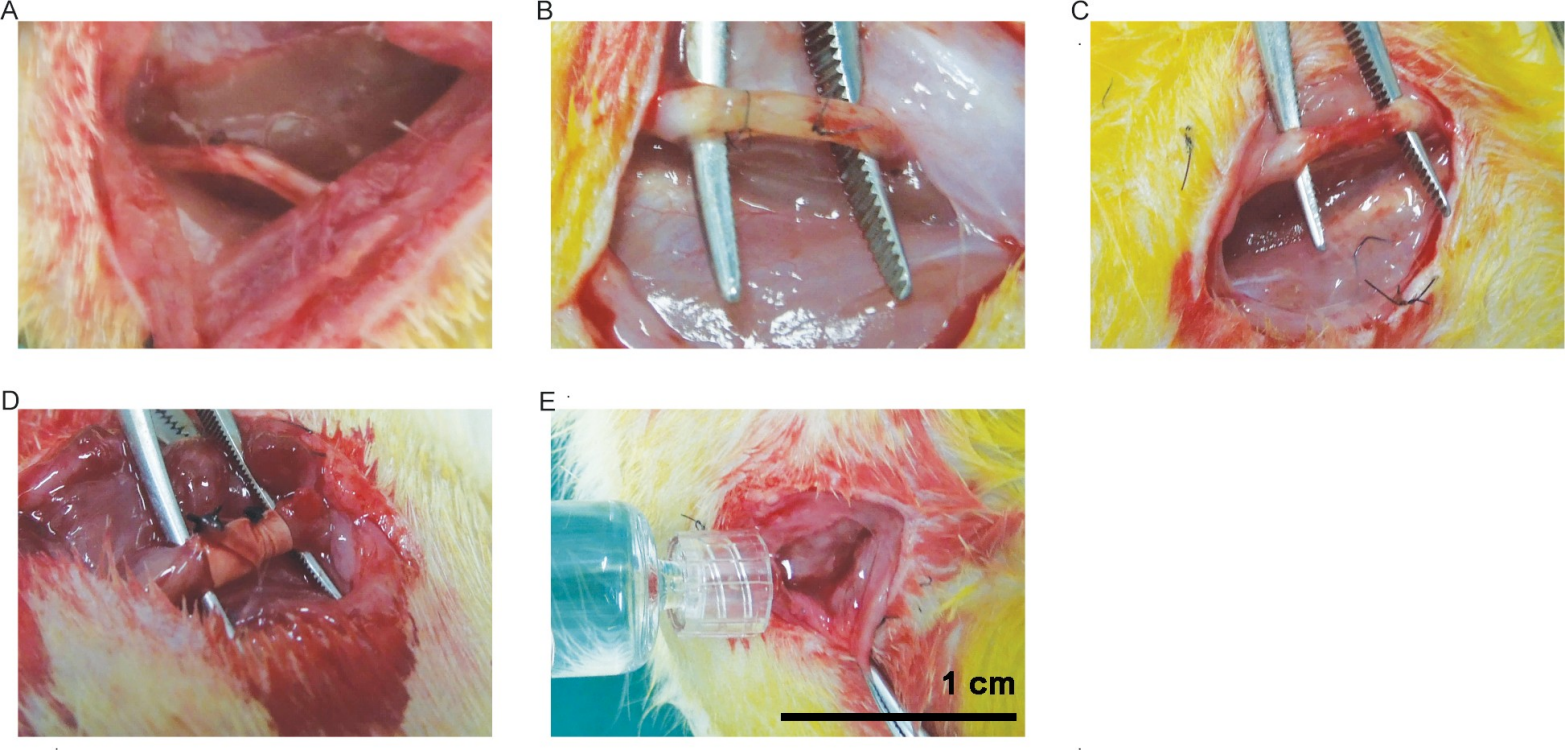
Rat model of sciatic nerve compression and treatment. **(A)** Normal sciatic nerve. **(B)** Silicone tube wrapped sciatic nerve. **(C)** The control group: Sciatic nerve after neurolysis. **(D)** The PCL-amnion group: Sciatic nerve was wrapped with PCL-amnion membrane. **(E)** The chitosan group: 0.5mL of chitosan hydrogel was injected into the compressed part.

Rats were anesthetized by intraperitoneal injection of 1% pentobarbital sodium (30mg/kg). A longitudinal incision was made in the dorsal skin of the right lower limb along the femur under sterile conditions. The sciatic nerve was exposed and wrapped by a silicone tube (6 mm in length, 1.2 mm in inner diameter and 2 mm in outer diameter) about 0.5 cm from the inferior border of the piriformis and then sutured with 7/0 absorbable suture according to the rat model of sciatic nerve compression designed by MacKinnon [27]. After 4 weeks, the surface of the silicone tube was covered by connective tissue, and the nerves at both ends were thickened and formed neuroma-like lesions. The compressed nerves were identified as moderate to severe demyelination, indicating that the model was successfully established (Fig 1A and 1B).

### 2.3 Treatment and examination of the rats with sciatic nerve compression

Chitosan is a natural polysaccharide that has structural characteristics similar to glycosaminoglycans and seems to mimic the functional behavior of glycosaminoglycans. Due to the bacteriostatic and hemostatic properties of chitosan [28], a medical chitosan hydrogel (YISHENGTANG MEDICAL PRODUCT CO.LTD, Shijiazhuang, China) was developed for clinical applications. Therefore, it was used as a positive control in this study. A total of ninety rats were randomly assigned to the PCL-amnion group (n = 30), the chitosan group (n = 30), or the control group (n = 30). Then, only the silicone tubes were removed in all rats, while the epineurium of the compressed nerve was not released. No treatments were given to the rats in the control group (Fig 1C). In the PCL-amnion group, the entrapped part of the sciatic nerve was wrapped with a PCL-amnion membrane and both ends of the membrane were sutured (Fig 1D). In the chitosan group, 0.5mL of medical chitosan hydrogel was injected into the entrapped part (Fig 1E). All operations were performed by the same researcher and assistant to avoid deviation.

After the operation, the rats were fed separately in a single cage to ensure adequate diet, kept warm and moisture proofed. The wound was disinfected with iodophor once a day for three consecutive days, and the suture was removed 7 days after surgery. At 2, 4, 8 and 12 weeks after the operation, 7 rats in each group were taken to observe the wound healing, whether there were plantar ulcers or autophagy.

At each time point (2^nd^ week, 4^th^ week, 8^th^ week, 12^th^ week), seven rats in each group were measured the conduction velocity and amplitude of sciatic nerve with myoelectricity-evoked potential apparatus (Viking Quest, Nicolet, US). The stimulating electrode was at the proximal end of the sciatic nerve trunk with an intensity of 5 mA for 0.2ms. The recording electrode was at the distal end of the sciatic nerve stem, about 10 mm away from the stimulating electrode. Conduction velocity = distance/conduction time. Moreover, the rats were intraperitoneally injected with sodium pentobarbital at a dose of 800 mg/kg for euthanasia [29] and opened along the original incisions to obtain the tissue samples for further assessment. At the sacrifice time points, the sciatic nerve was re-exposed and adhesions scored according to Petersen et al. [30] by morphological analysis. Briefly, 1 point, skin, muscle, and fascia tissue are completely healed; 2 points are partially cracked; 3 points are completely cracked. The scoring standard for separating the regenerated nerve from the surrounding tissues: 1 point 1 requires no or only slight blunt dissection; 2 points 1 requires more blunt separation; 3 points 1 requires sharp separation.

### 2.4 Histological and immunohistochemical assessment

About 2 mm of nerve segments was obtained from two rats in each group on week 2, 4, 8, 12 for histological observation. Nerve segments were fixed in 10% formalin solution and stained with hematoxylin and eosin (HE).

To observe the process of nerve regeneration, Schwann cells were labeled with anti-S-100 protein and observed under a light microscope. Anti-CCR7 (rabbit immunoglobulin G, #ab253187, 1:100, Abcam, MA, USA) was used to label the pro-inflammatory M1 macrophage for observation of inflammatory reaction in the nerve scar tissues.

To observe the repair of never fibers, the segments were fixed in 3% glutaraldehyde and embedded in epoxy resins. Ultra-thin cross-sections were cut on an ultramicrotome and observed with transmission electron microscopy (TEM).

### 2.5 Western blot

Proteins extracted from compressed sciatic nerve segments of five rats in each group on week 2, 4, 8, 12 were separated by SDS-PAGE and then transferred to polyvinylidene difluoride (PVDF) membranes. After blocked by 5% non-fat milk, the membranes were incubated with the antibodies of anti-type I collagen (COL-I, #ab6308, 1:1000, Abcam, MA, USA) and anti-type III collagen (COL-III, #BE3163, 1:1000, EasyBio, Beijing, China), respectively. After washed with PBST, the membranes were incubated with horseradish peroxidase (HRP)-labeled anti-mouse polyclonal antibody (1:2400). Target bands were analyzed with ImageJ software to evaluate the levels of type I and type III collagen by calculating the ratio of collagen absorbance to β—actin absorbance.

### 2.6 RT-PCR

At each time point (2^nd^ week, 4^th^ week, 8^th^ week, 12^th^ week), the total RNA from 3 mm compressed sciatic nerve of five rats in each group were extracted using TRIzol reagent according to the product description. The quantity of RNA was detected using NanoDrop® ND-2000. After applied to agarose gel electrophoresis, the RNA was reverse transcripted to cDNA and amplified with PCR amplification instrument (denaturation at 95°C for 30s, annealing at 60°C for 30s, and elongation at 72°C for 30s, 40 cycles in total). Glyceraldehyde phosphate dehydrogenase (GAPDH) was used as endogenous control and the primer sequences were shown in Table 1. PCR products were analyzed by ΔΔCT method [31].

**Table 1:** Primer sequences.

Primer name	Sequence 5 '- 3'	Product length
**NGF-F**	CGGTCTTCCCGCCCTAGCCTG	132
**NGF-R**	ATTTGCACGCCGCTCCTTTGC	
**GAPDH-F**	CGTGTTCCTACCCCCAATGT	83
**GAPDH-R**	TGTCATCATACTTGGCAGGTTTCT	

### 2.7 Statistics

SPSS 21.0 software was used for data processing. All data were expressed as mean ± standard deviation (SD) and analyzed by one-way ANOVA. P < 0.05 was significant difference. Kruskal-Wallis test and hoc-Bonferroni test were used to evaluate nerve adhesion.

## 3. Results

### 3.1 General observation of rat model

**Fig 2:**
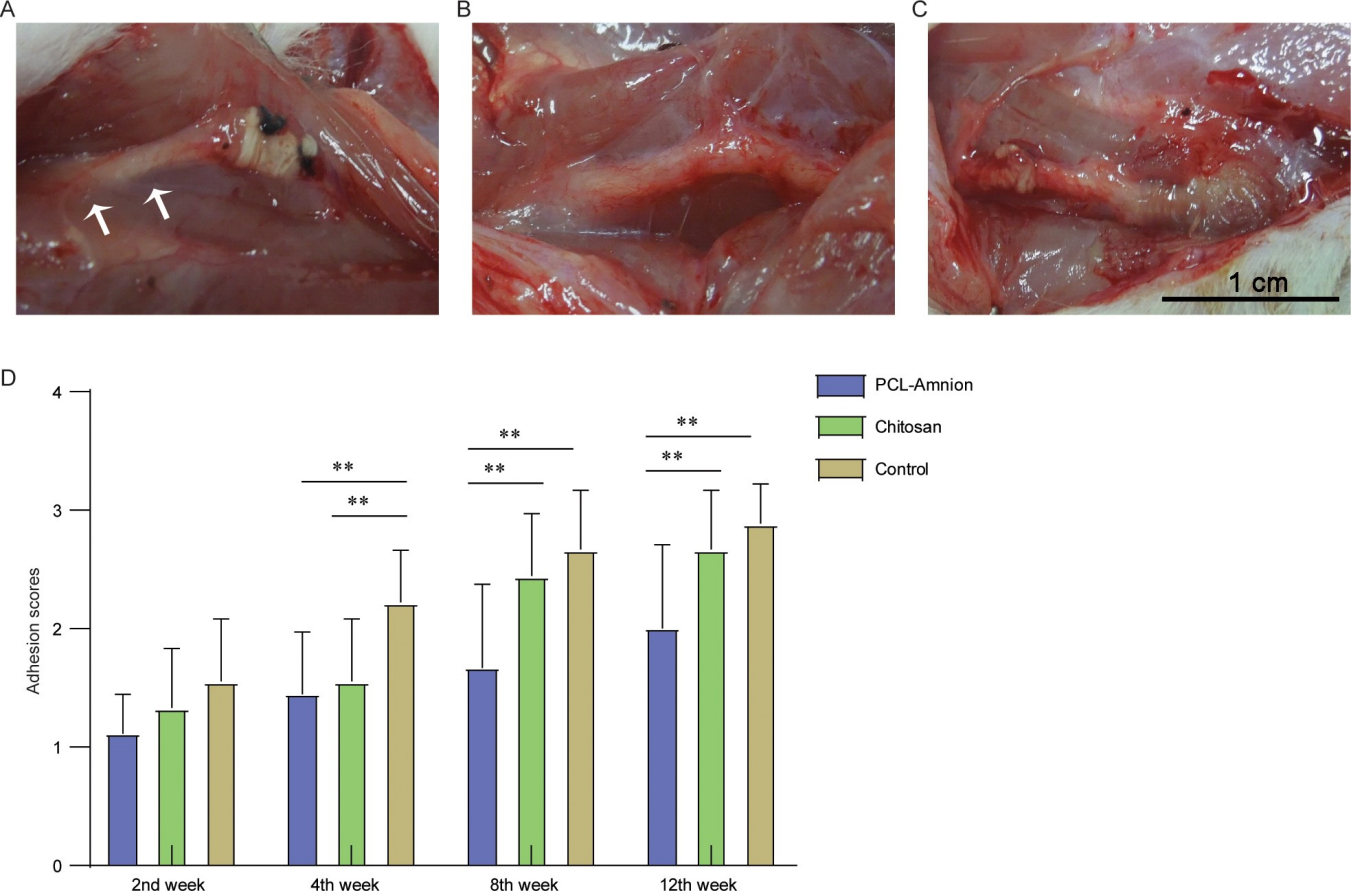
Adhesion assessment of the three groups at the 12th week. **(A)** The PCL-amnion group. **(B)** The chitosan group. **(C)** The control group. **(D)** Adhesion scores of the three groups at each time point. **P<0.05.

No infection, foot ulcer or autophagy were found in all rats in three groups. Two weeks after the operation, the subcutaneous tissue and peripheral nerve tissue of rats were slightly congested and edematous. The compressed nerves became thinner and the neuroma-like lesions at both ends decreased. In the PCL-amnion group and chitosan group, loose adhesions were found around the nerve, while in the control group, loose adhesions were extensive, which was beneficial to blunt separation. Four weeks after the operation, there were more extensive and dense adhesions found in the control group compared with the PCL-amnion and chitosan group. Eight weeks after the operation, in the PCL-amnion group, there was no obvious hyperemia and edema and the compressed segments were significantly thickened, while the chitosan was completely absorbed. In the control group, congestion and dense adhesion of epineurium were observed. Twelve weeks after the operation, the congestion of epineurium disappeared in all groups. In the PCL-amnion group (Fig 2A), the PCL-amnion membranes were partially absorbed, and the loose adhesions were easy for blunt separation. However, the extensive and dense adhesions were hard to separate in the chitosan group (Fig 2B) and the control group (Fig 2C). The degree of adhesion was evaluated using the scoring system proposed by Petersen *et al* [30], and the results showed that there was no significant difference among the three groups in the first two weeks (*P*>0.05). In the fourth week, the scores of the PCL-amnion group and chitosan group were lower than those of the control group. At the 8th and 12th week, the scores of the PCL-amnion group were significantly lower than those of the chitosan group and control group (*P*<0.05) (Fig 2D).

### 3.2 Nerve electrophysiological assessment

**Fig 3:**
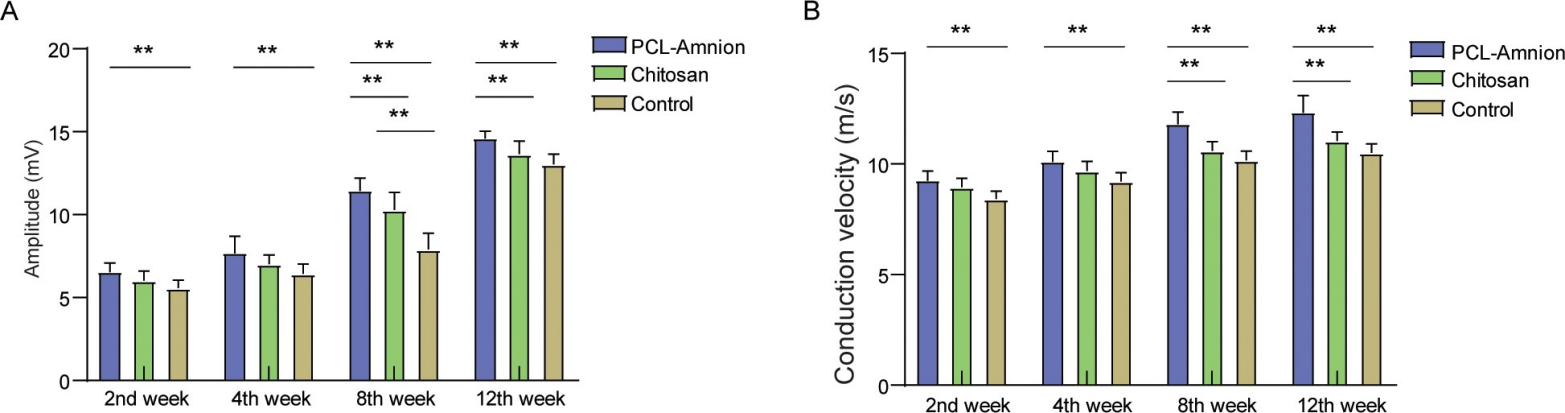
Electrophysiological assessment of the three groups at each time point. **(A)** Comparison of amplitude of nerve conduction. **(B)** Comparison of conduction velocity. **P<0.05.

As shown in Fig 3, the amplitude and velocity of nerve conduction in the three groups gradually increased with time. The amplitude and conduction velocity of the PCL-amnion group were always significantly higher compared with the control group (*P*<0.05). There was no significant difference in amplitude and conduction velocity between the PCL-amnion group and the chitosan group at the second and fourth weeks. At week 8 and week 12, the amplitude and conduction velocity of the PCL-amnion group were significantly higher than those of the chitosan group (*P*<0.05).

### 3.3 Evaluation of nerve regeneration

**Fig 4:**
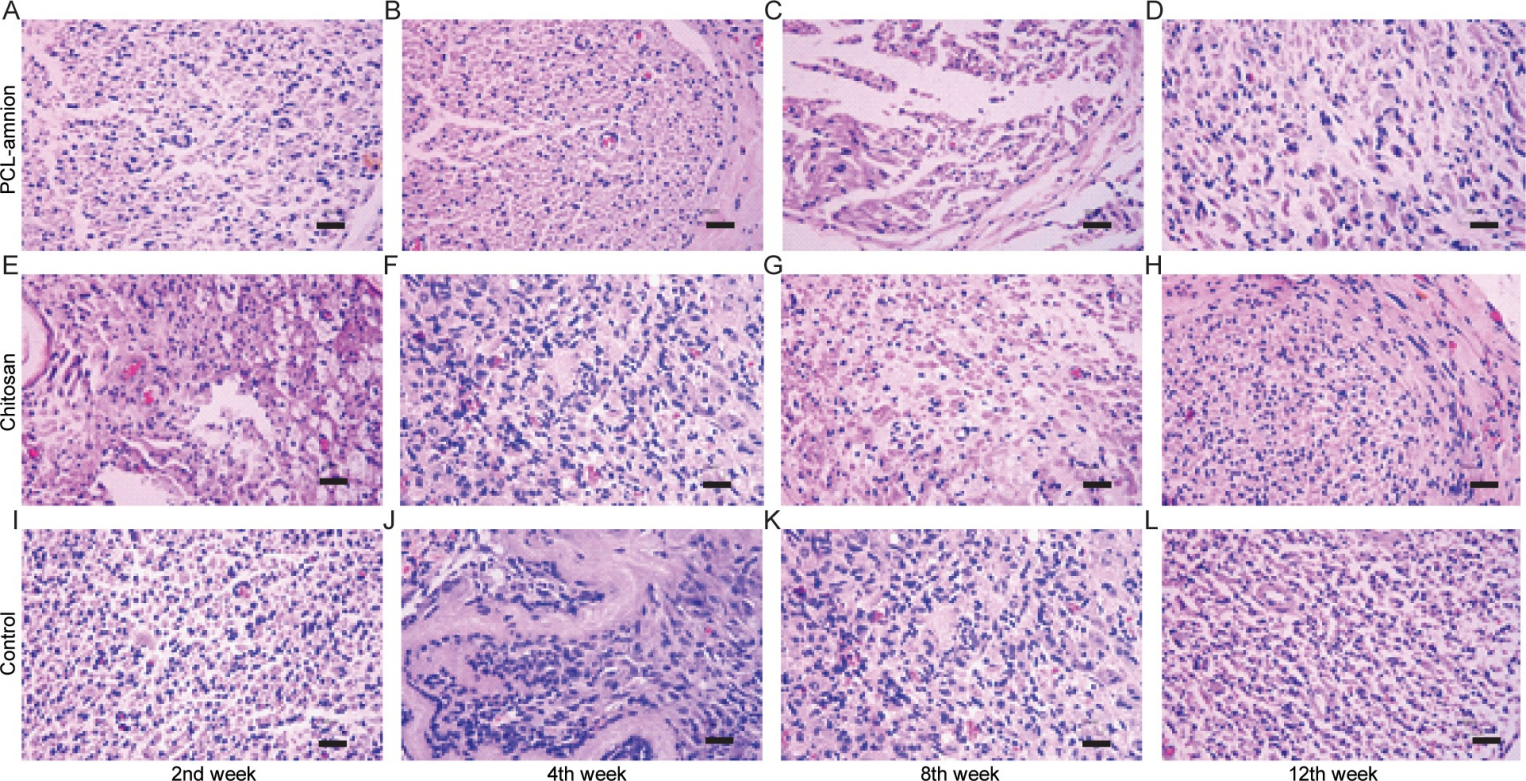
H&E staining of nerve tissue in the three groups at each time point (x400). **(A-D)** The PCL-amnion group. **(E-H)** The chitosan group. **(I-L)** The control group. Scale bar = 50 μm.

**Fig 5:**
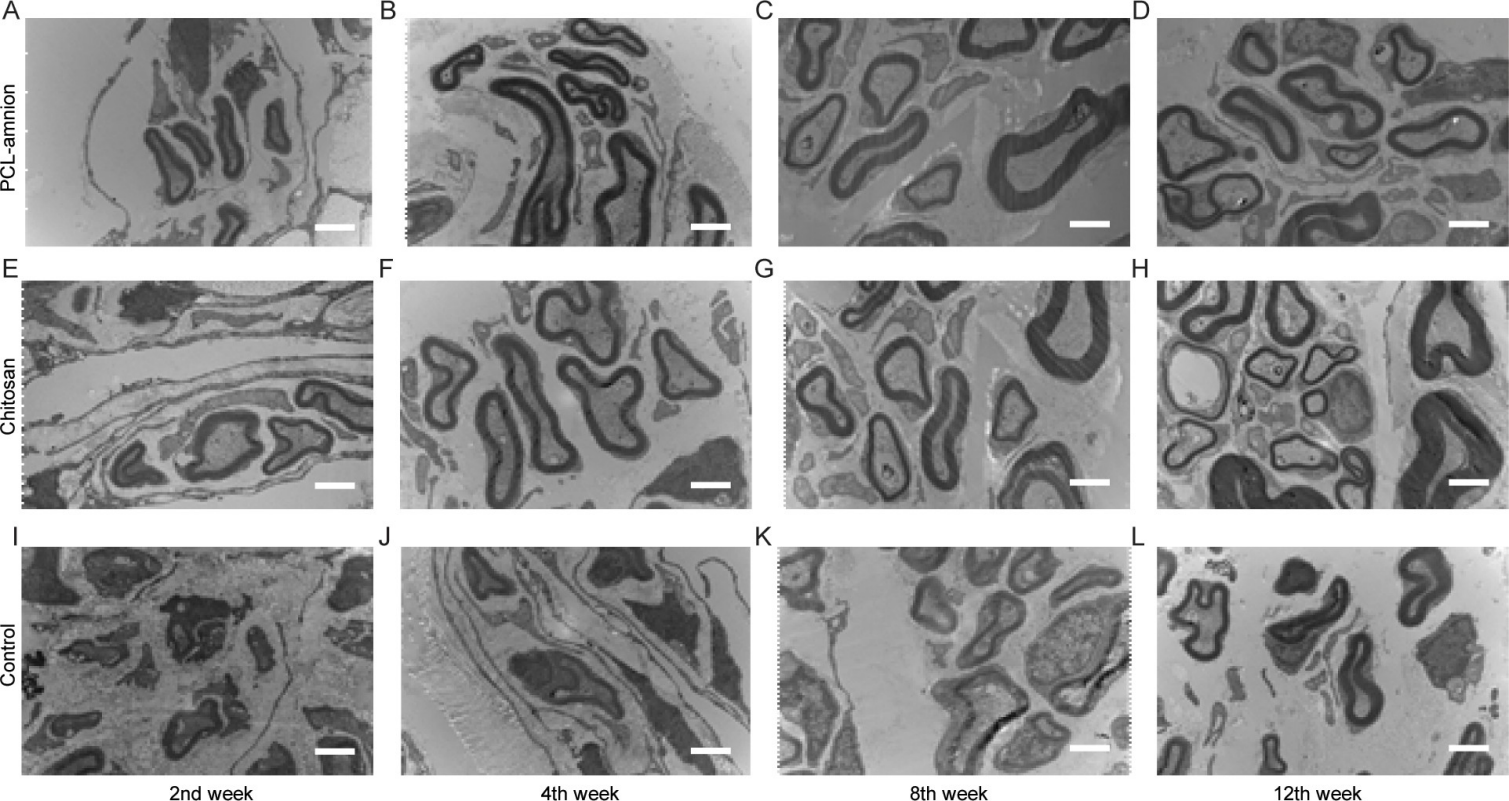
Transmission electron microscope images of the nerve tissues at each time point (x10000). **(A-D)** The PCL-amnion group. **(E-H)** The chitosan group. **(I-L)** The control group. Scale bar = 2 μm.

**Fig 6:**
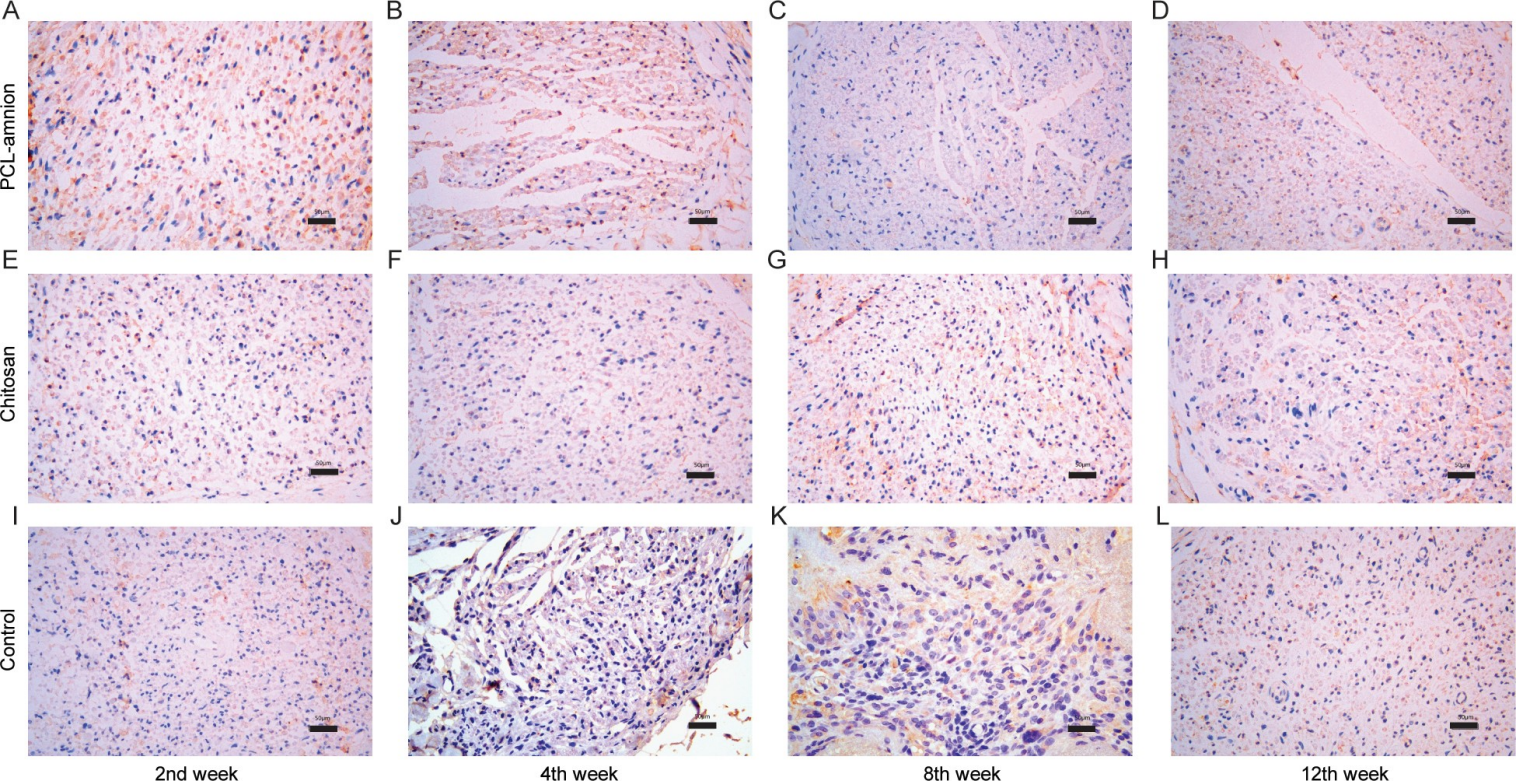
Proliferation of Schwann cells and expression of S-100 protein in the three groups at each time point (x400). From week 2 to week 8, the number of Schwann cells and the expression of S-100 in the PCL-amnion group **(A-C)** were higher than those in the chitosan group **(E-G)** and control group **(I-K)**. At the 12th week, the number of Schwann cells and expression of S-100 in all three groups decreased, but those in the PCL-amnion group **(D)** were still higher compared with the chitosan group **(H)** and control group **(L)**. Scale bar = 50 μm.

To evaluate the nerve regeneration, we observed the nerve tissues by H&E staining and transmission electron microscope. In the second week, the epineurium structures were intact in all groups, but there were different degrees of demyelination. A large amount of inflammatory cells infiltration around the nerve fibers. In the PCL-amnion group, inflammatory cells were confined to the superficial layer of the epineurium (Fig 4A). In the chitosan group (Fig 4E) and control group (Fig 4I), more inflammatory cells and scattered multinucleated giant cells were observed. TEM images showed that there was axonal sprouting around the compressed section in the PCL-amnion group and chitosan group, which was surrounded by perineurial cells with a few mitochondria, microfilaments, microtubules, and vesicles (Fig 5A and 5E). In the control group, swelling of the myelin sheath, separation of basement membrane and axon, and mitochondrial necrosis were observed (Fig 5I). In the 4th week, the inflammatory reaction and myelin sheath swelling were alleviated in all rats. Compared with the control group (Fig 4J), there were fewer inflammatory cells in the PCL-amnion group (Fig 4B) and chitosan group (Fig 4F). In the PCL-amnion group (Fig 5B), the myelin sheath was thicker than that of the chitosan group (Fig 5F) and regular regenerated axons with organelles were observed. However, there were a small amount of irregularly arranged axons and limited myelin sheath disintegration in the control group (Fig 5J). At the 8th week, demyelination was significantly alleviated in all groups. There were more regenerated nerve fibers with thin myelin in the PCL-amnion group (Fig 4C) and chitosan group (Fig 4G) compared with the control group (Fig 4K). TEM images presented significant myelinated fibers regeneration in the PCL-amnion group (Fig 5C) and chitosan group (Fig 5G). In the PCL-amnion group, myelinated fibers were arranged orderly, myelin sheath was thickened, and no connective tissue hyperplasia was found. In the control group, the diameter of axons became smaller and connective tissue hyperplasia was observed (Fig 5K). At the 12th week, there were abundant regenerated axons and thick myelin formed in the PCL-amnion group (Fig 4D). As shown in the TEM image (Fig 5D), the nerve fibers were surrounded by intact myelin and Schwann cells with normal cellular structure. A small number of myelinated fibers were assembled into bundles. In the chitosan group (Fig 4H) and control group (Fig 4L), the surface of the nerves was not as smooth as the PCL-amnion group. Moreover, although nerve fibers were orderly arranged, the diameter and myelin were thinner and the wrapped perineurium was less compact than that in the PCL-amnion group. TEM images showed that mitochondria of the chitosan group (Fig 5H) and control group (Fig 5L) were swollen and cristae were not clear (Fig 5L). Immunohistochemical results showed that the proliferation of Schwann cells and the expression of S-100 in the PCL-amnion group were higher than those in the chitosan group and control group over time (Fig 6).

### 3.4 Inflammatory reaction in the process of nerve regeneration

**Fig 7:**
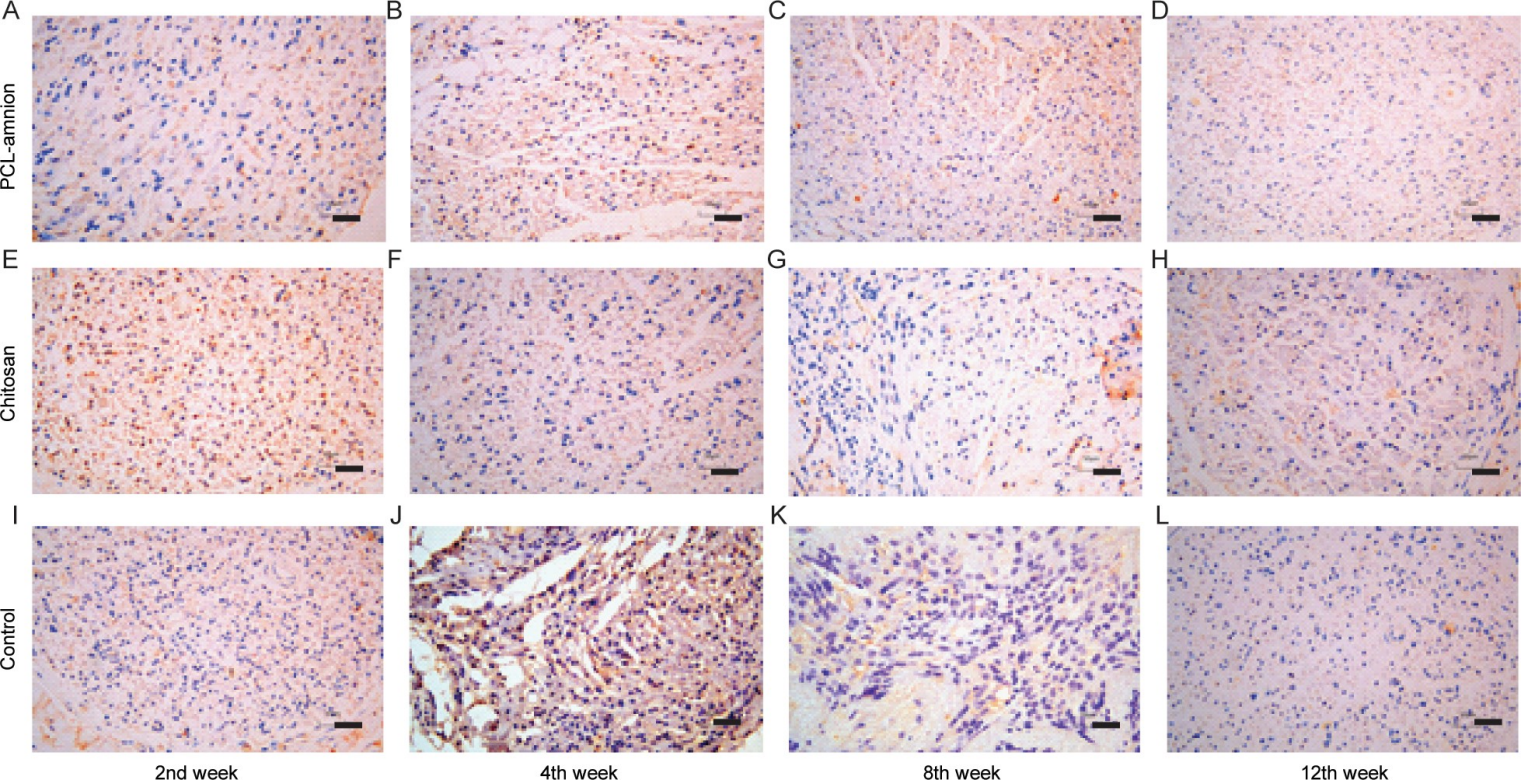
Immunohistochemical results of CCR7-positive M1 macrophages at each time point (x400). **(A-D)** The PCL-amnion group. **(E-H)** The chitosan group. **(I-L)** The control group. Scale bar = 50 μm.

To evaluate the inflammatory response during nerve regeneration, M1 macrophages were labeled with the anti-CCR7 antibody. As shown in Fig 7, the number of CCR7-positive cells decreased in all three groups over time. Moreover, the number of M1 macrophages in the PCL-amnion group was less than that in the chitosan group and control group at each time point.

**Fig 8:**
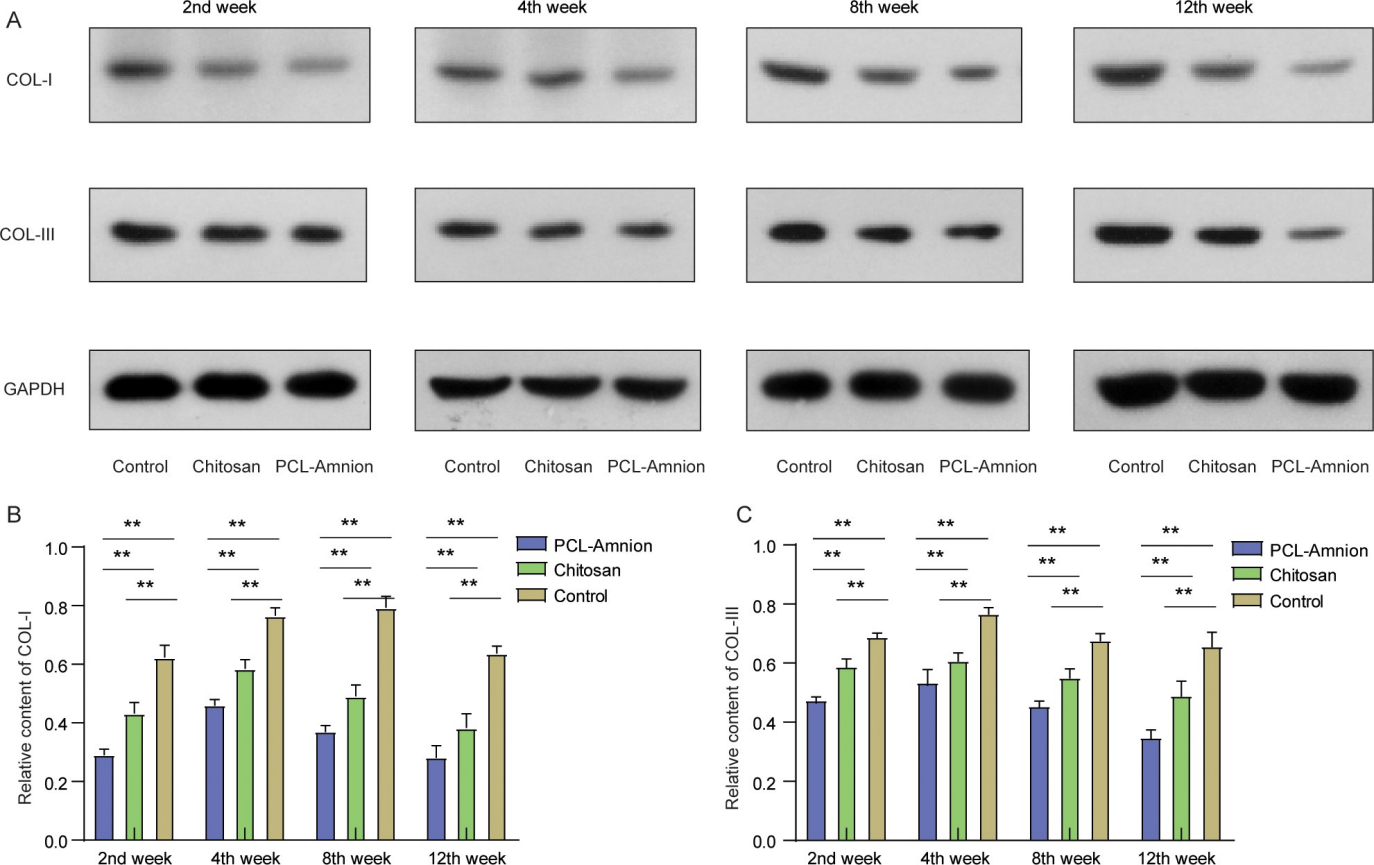
The relative contents of COL-I and III at each time point. **(A)** Expressions of COL-I and COL-III were detected by western blot. **(B)** The relative contents of COL-I were compared among the three groups. **(C)** The relative contents of COL-III were compared among the three groups. **P < 0.01.

To examine the deposition of fibrin, we detected the expression levels of COL-I and COL-III in the compressed nerves. As shown in Fig 8, the relative contents of COL-I (Fig 8B) and COL-III (Fig 8C) in each group increased with time. The relative content of COL-I reached a peak in week 8, and the relative content of COL-Ⅲ was the highest in week 4, and then the amount of them decreased. At each time point, the expression levels of COL-I and COL-III in the PCL-amnion group were all significantly lower than those in the chitosan group and the control group (*P* < 0.05).

### 3.5 PCL-amnion membrane might repair never by releasing nerve growth factor

**Fig 9:**
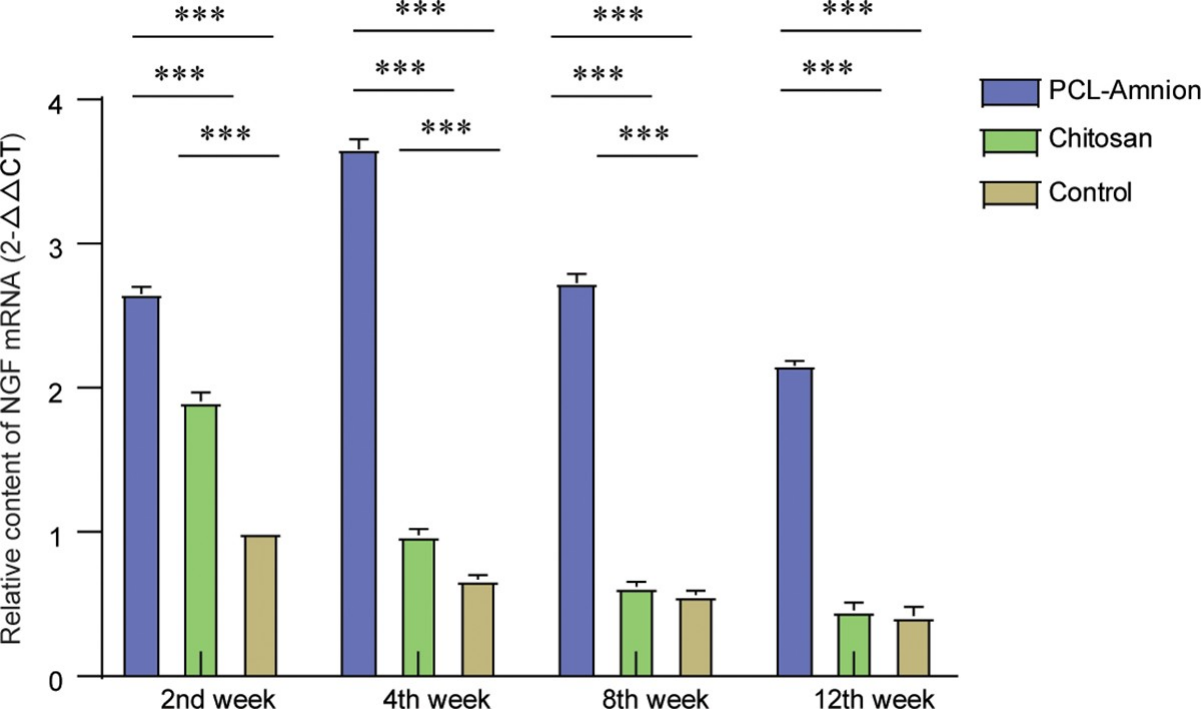
Comparison of the expression levels of NGF mRNA at each time point. **P<0.05.

As shown in Fig 9, the relative content of the mRNA expression of nerve growth factor (NGF) in the chitosan group and control group decreased gradually with time. The content of NGF mRNA in the PCL-amnion group reached the peak at the 4th week, which was significantly higher compared with the other two groups (*P* < 0.05). After 8 weeks. there was no significant difference between the chitosan group and control group (*P* > 0.05).

## 4. Discussion

The ideal materials for nerve repair should inhibit inflammation, prevent nerve adhesion and scarring, and promote nerve growth [32]. Although PCL has been widely used as an artificial biomaterial due to its plasticity, biodegradability, and good mechanical properties, its hydrophobicity and poor cell affinity limit its clinical application [33]. Human amniotic epithelial cells have characteristics similar to those of mesenchymal stem cells, that is, they have the potential to differentiate into multiple lineages [34]. Considered postpartum waste, the application of human amniotic membranes is not ethically controversial. To combine the advantages of the two materials, we spinned PCL onto the surface of an amniotic membrane using electrospinning. The PCL-amnion nanofibrous membrane had better mechanical strength and biocompatibility. In a previous study, we found that an electrospun PCL-amnion nanofibrous membrane effectively prevented post-surgical tendon adhesion and promoted tendon healing [25]. Although there is a number of studies that report on the application of amniotic membranes in nerve repair, they are not yet widely used in the clinic [24, 35]. Therefore, we investigated the effects of PCL-amnion on the repair of sciatic nerve compression.

The process of peripheral nerve repair and regeneration is affected by multiple factors, such as the surrounding microenvironment, in which local scar adhesion is particularly important [36]. Fibroblasts and inflammatory cells enter the impaired nerve and induce fibrosis and scarring, thereby leading to new compression [37]. In the current study, the adhesion scores indicated that the adhesion of compressed sciatic nerves was significantly inhibited after treatment with PCL-amnion, and the treatment effect of PCL-amnion was more persistent than that of chitosan. We also applied electromyography, to evaluate the recovery of nerve function more objectively. We found no significant differences in conduction velocity between the three groups. However, after 4 weeks, the functional improvement after PCL-amnion treatment was more significant than that after control or chitosan treatment. In addition, fewer inflammatory reactions and less tissue adhesion were found after PCL-amnion treatment. Tissue adhesion usually occurs 1–3 months after neurosurgery [38]. The PCL-amniotic membrane might build a protective sheath around the impaired nerve, which separates the regenerated from the surrounding tissues. Studies have also shown that the adhesion resistance of the amniotic membrane is not only related to a mechanical barrier effect but also the secretion of growth factors and immune regulatory factors [39]. The axon images presented in the current study show that the nerve fibers in PCL-amnion treated rats were arranged regularly with fewer collagen fibers. Compared with the chitosan-treated rats and the control group, the myelin sheath was thicker and more mature in the PCL-amnion rats, consistent with the fact that the cytokines secreted from the amnion accelerated the repair of the nerves.

After the disintegration of the axon and the myelin sheath, the permeability of the vascular-nerve barrier increases significantly [40]. The released Schwann cells, which are glial cells around the axon, proliferate rapidly and form Büngner bands along the basement membrane that promotes nerve regeneration [41]. S-100 protein (a marker of Schwann cells) was highly expressed in the PCL-amnion rats compared with the chitosan-treated rats and control group after 4 weeks, indicating the enhanced proliferation of nerve cells. Moreover, inflammatory responses induced by the hemorrhage during neurolysis and the damage of the nerves and the surrounding tissues lead to enhanced capillary permeability, macrophage migration, and fibrin deposition [42]. Amniotic membranes have been reported to reduce the inflammatory response by inhibiting the expression of pro-inflammatory cytokines such as interferon-C, interleukin (IL)-1, IL-2, IL-8, IL-10, tumor necrosis factor-β, and platelet-derived growth factor [43]. To evaluate the inflammation and potential immune response that occur in the local scarring area, we labeled the pro-inflammatory M1 macrophage with CCR7 antibody. Immunohistochemistry results showed a large amount of inflammatory cells infiltrating the area around the nerve fibers in all three groups. Fewer CCR7-positive cells were counted in the PCL-amnion rats than in the chitosan-treated rats and control group at each time point, which implies that PCL-amnion effectively reduced the inflammatory response by preventing the invasion of macrophages. These results also show the low immunogenicity of PCL-amnion. Moreover, the expression levels of COL-I and COL-III were significantly lower in the PCL-amnion-treated rats than in the chitosan-treated rats and control group, suggesting that PCL-amnion played a role in reducing the deposition of fibrin and protecting the regenerated nerve from infiltrating scar tissues.

Amniotic membranes are known to secrete many kinds of neurotrophic factors such as NGF, brain-derived neurotrophic factor (BDNF), neurotrophin-3, glia-derived neurotrophic factor (GDNF), and ciliated neurotrophic factor (CNTF) [44]. However, these factors degrade rapidly *in vivo* and cannot be maintained at therapeutic doses [45]. NGF plays a key role in promoting the growth and development of the nervous system. NGF can protect the neurons from further damage in the already impaired nervous system, promotes the regeneration of nerve fibers and axons, improves nerve conduction function, and relieves neuropathic pain by restoring the integrity of the myelin sheath [46]. Preclinical studies have shown that levels of NGF are increased in preclinical models of inflammation and peripheral nerve injury [47]. In patients with peripheral neuropathy caused by chemotherapy, the decrease in circulating NGF levels was associated with the severity of their neuropathy [48]. Moreover, local injection of microspheres capable of controlling NGF release promoted regenerating peripheral nerves to cross the nerve gap [49]. NGF levels can therefore be used as an indicator of neural repair [50, 51]. We found that the expression levels of NGF were significantly higher in the PCL-amnion-treated rats than in the chitosan-treated rats and control group at each time point, indicating the advantage of amniotic membranes over non-biological materials in nerve repair. After 4 weeks, NGF levels had decreased in all three groups, but in the PCL-amnion-treated rats, high NGF levels were maintained longer than in the other two groups. We speculate that this might be related to the formation of stable and relatively closed PCL-amniotic membrane microchambers, in which concentrated Schwann cells and a variety of neuroactive factors are maintained and repair continues.

## 5. Conclusion

In conclusion, coating the surface of an amniotic membrane with PCL by electrospinning compensated for the shortcomings of easy folding and hard to stitch, and prolonged the maintenance of the amnion *in vivo*. PCL-amnion effectively alleviated the adhesion and inflammation of nerve tissue in the convalescent period. Moreover, the microenvironment created by PCL-amnion may enhance the proliferation of Schwann cells and increase the level of nerve growth factors, which can promote nerve regeneration. The repair effect of PCL-amnion on other types of nerve injury and its potential clinical applications need to be further investigated in follow-up studies.

## Supporting information

S1 File (PDF)
