# Correction: Electrospun polycaprolactone (PCL)-amnion nanofibrous membrane prevents adhesions and promotes nerve repair in a rat model of sciatic nerve compression

**DOI:** 10.1371/journal.pone.0253699

**Published:** 2021-06-17

**Authors:** Ruiyi Dong, Chunjie Liu, Siyu Tian, Jiangbo Bai, Kunlun Yu, Lei Liu, Dehu Tian

A second affiliation is missing for the first author. Ruiyi Dong is also affiliated with Department of Hand Surgery, The Third Affiliated Hospital of Hebei Medical University, Shijiazhuang, Hebei, 050051, China.
