# Supplementary material for: Electrospun polycaprolactone (PCL)-amnion nanofibrous membrane prevents adhesions and promotes nerve repair in a rat model of sciatic nerve compression
Source: PLoS One. 2020 Dec 18;15(12):e0244301. doi: 10.1371/journal.pone.0244301 (PMC7748280; doi:10.1371/journal.pone.0244301)
Supplement: S1 File — (PDF) [file pone.0244301.s001.pdf]

Original Image

Original Image of PCR

Melt Curve and Application of gene NGF

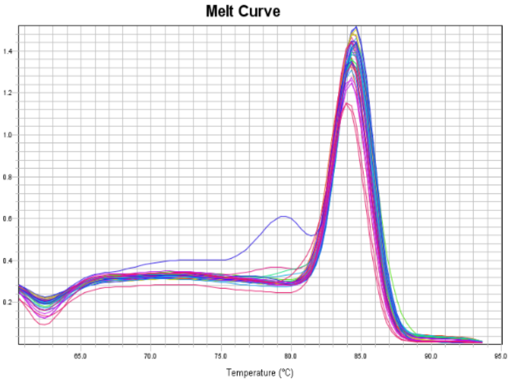

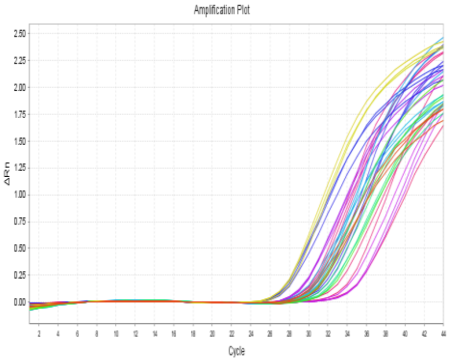

Melt Curve and Application of gene GAPDH

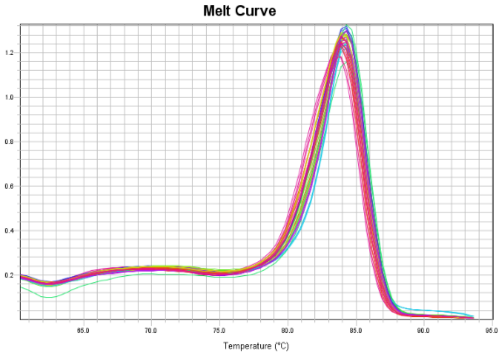

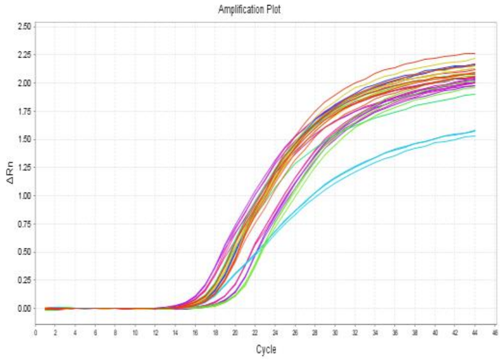

Original Image of WB

Results of protein CollagenI, CollagenIII and GAPDH in 2 weeks

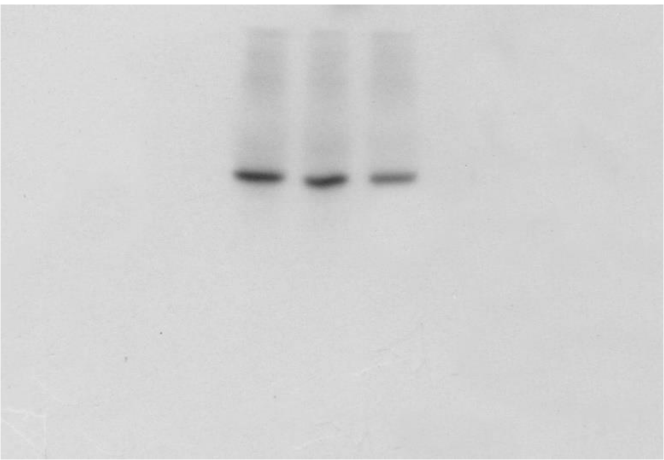

CollagenI-2W

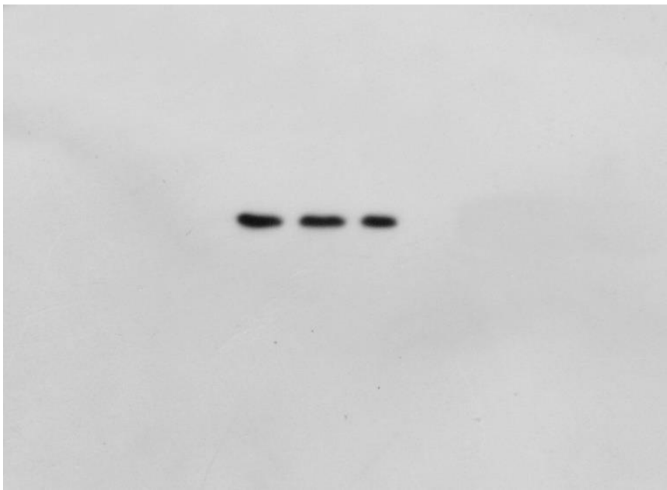

CollagenIII-2W

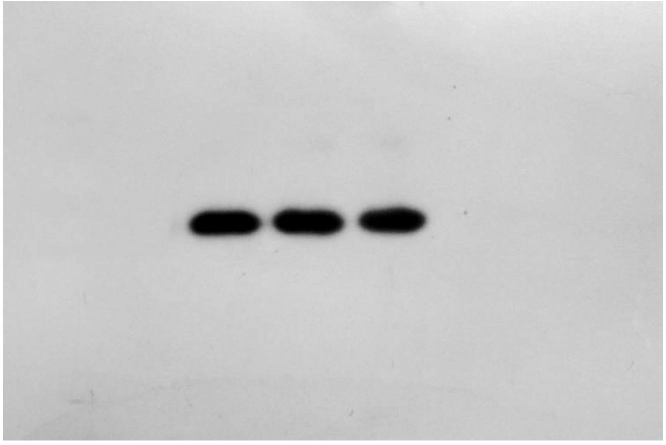

GAPDH-2W

Results of protein CollagenI, CollagenIII and GAPDH in 4 weeks

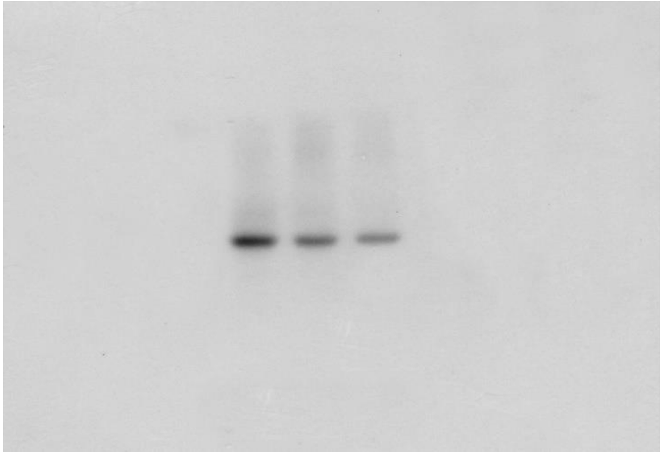

CollagenI-4W

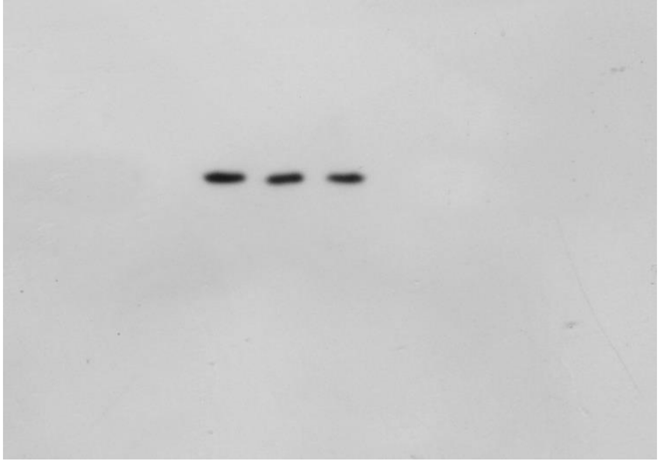

CollagenIII-4W

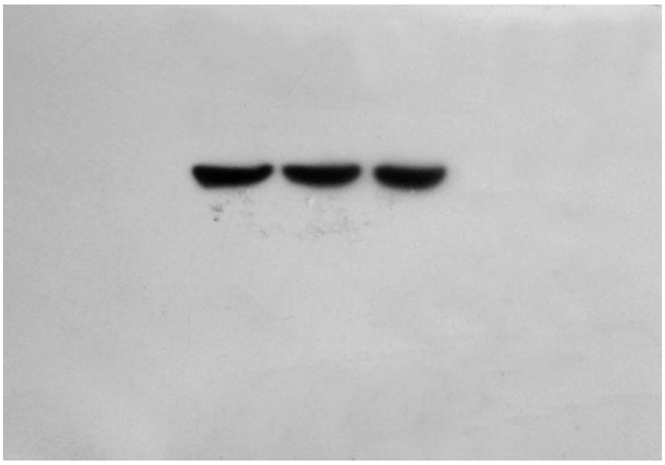

GAPDH-4W

Results of protein CollagenI, CollagenIII and GAPDH in 8 weeks

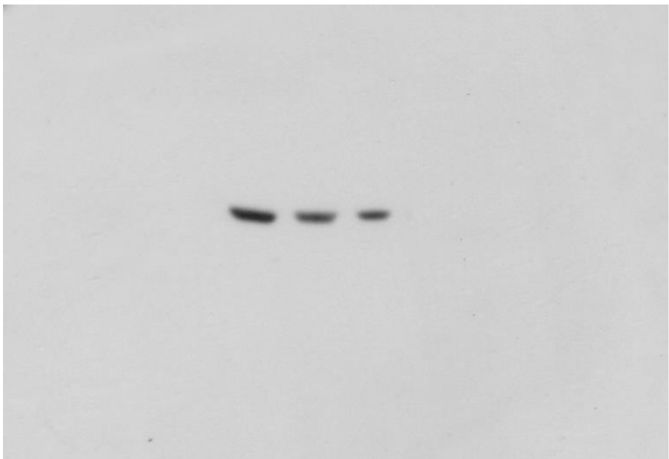

CollagenI-8W

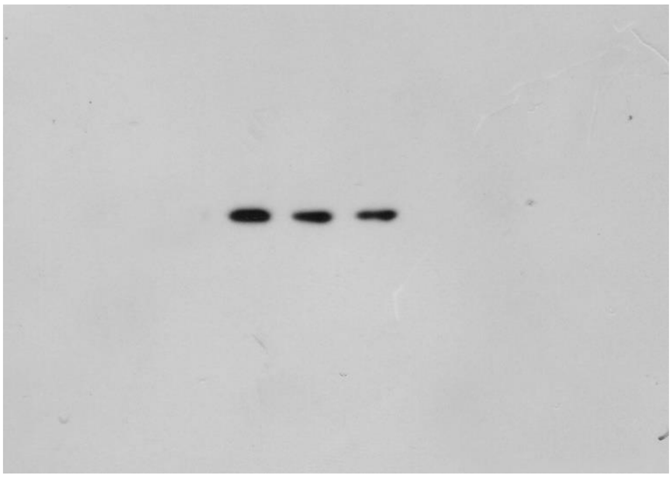

CollagenIII-8W

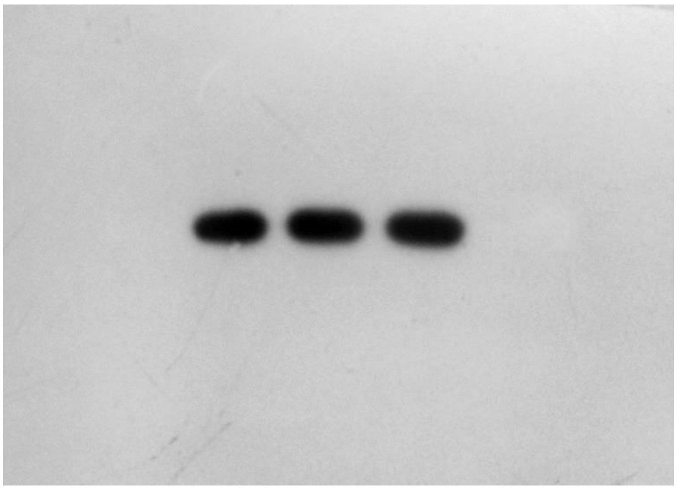

GAPDH-8W

Results of protein CollagenI, CollagenIII and GAPDH in 12 weeks

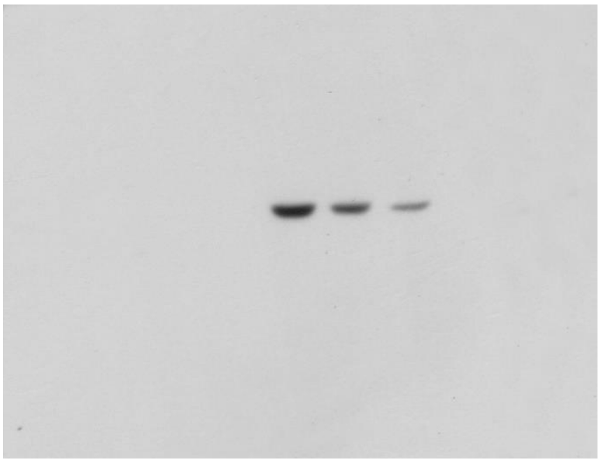

CollagenI-12W

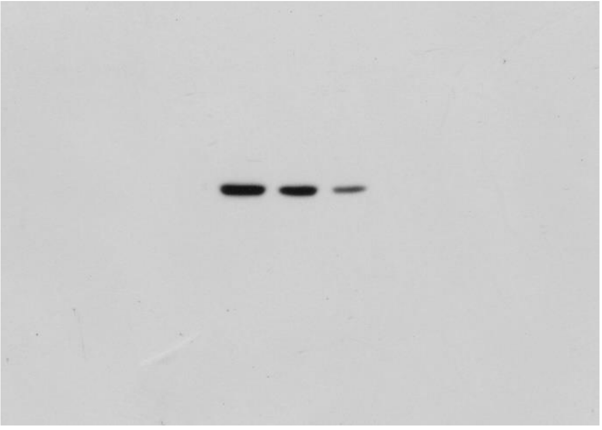

CollagenIII-12W

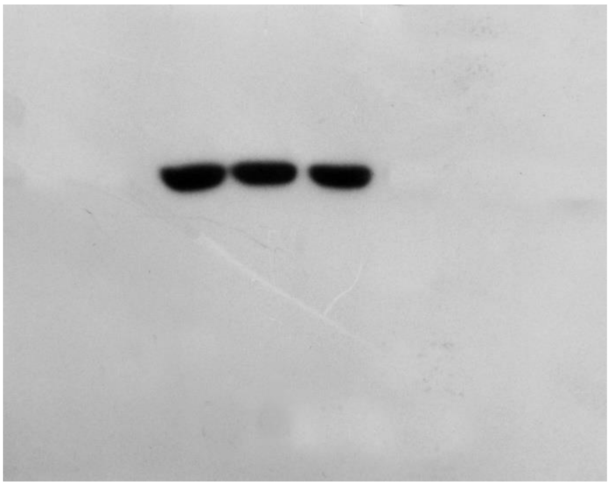

GAPDH-12W

Original Image of Electromyography

Because the image is too large, we put the original picture on the network disk, could be viewed by the website and code as follow:

website： https://pan.baidu.com/s/1Q-3yCEKPk6TrMyp56jWDMQ

code： 3vfg

Original Image of HE Staining

Because the image is too large, we put the original picture on the network disk, could be viewed by the website and code as follow:

website： https://pan.baidu.com/s/1F9GsutXuhv3znxuQ0KSDmw

code： fc62

Original Image of Immunohistochemistry

Because the image is too large, we put the original picture on the network disk, could be viewed by the website and code as follow:

website: https://pan.baidu.com/s/1Y4CK_sc3AY_rnzjQIOipOw

code: ev87
